# Dual role of YM1+ M2 macrophages in allergic lung inflammation

**DOI:** 10.1038/s41598-018-23269-7

**Published:** 2018-03-23

**Authors:** Christina Draijer, Patricia Robbe, Carian E. Boorsma, Machteld N. Hylkema, Barbro N. Melgert

**Affiliations:** 1 Department of Pharmacokinetics, Toxicology and Targeting, University of Groningen, Groningen, The Netherlands; 2 GRIAC- Groningen Research Institute for Asthma and COPD, University Medical Center Groningen, University of Groningen, Groningen, The Netherlands; 3 Department of Pathology, University Medical Cente Groningen, University of Groningen, Groningen, The Netherlands

## Abstract

Alternatively activated (M2 or YM1+) macrophages have been associated with the development of asthma but their contribution to disease initiation and progression remains unclear. To assess the therapeutic potential of modulating these M2 macrophages, we have studied inhibition of M2 polarisation during and after development of allergic lung inflammation by treating with cynaropicrin, a galectin-3 pathway inhibitor. Mice that were treated with this inhibitor of M2 polarisation during induction of allergic inflammation developed less severe eosinophilic lung inflammation and less collagen deposition around airways, while the airway α-smooth muscle actin layer was unaffected. When we treated with cynaropicrin after induction of inflammation, eosinophilic lung inflammation and collagen deposition were also inhibited though to a lesser extent. Unexpectedly, both during and after induction of allergic inflammation, inhibition of M2 polarisation resulted in a shift towards neutrophilic inflammation. Moreover, airway hyperresponsiveness was worse in mice treated with cynaropicrin as compared to allergic mice without inhibitor. These results show that M2 macrophages are associated with remodeling and development of eosinophilic lung inflammation, but prevent development of neutrophilic lung inflammation and worsening of airway hyperresponsiveness. This study suggests that macrophages contribute to determining development of eosinophilic or neutrophilic lung inflammation in asthma.

## Introduction

Allergic asthma is a heterogeneous disorder of the lungs and those affected suffer from symptoms such as wheezing, coughing and chest tightness. These symptoms are caused by lung inflammation, airway hyperresponsiveness (AHR), mucus hypersecretion, and airway wall remodeling, often due to exposure to allergens such as house dust mite (HDM). The lung inflammation is characterized by increased infiltration of Th2 lymphocytes, production of Th2 cytokines interleukin(IL)-4, IL-13, and IL-5, and increased numbers of mast cells, eosinophils and macrophages^1^.

What kind of role macrophages play in asthma has been the subject of much debate recently. On the one hand studies have shown macrophages to be important in maintaining lung homeostasis and protecting against inflammation^2–6^, but others studies, including our own, have shown that macrophages can contribute to development and severity of asthma^5,7–14^. These different findings may be due to the plastic behavior of macrophages. Macrophages can adopt many different polarisation states depending on the signals they receive from tissues. These polarisation states range from a proinflammatory state with expression of interferon regulatory factor 5 (IRF5, also known as classically activated or M1 macrophages) to a prorepair state with expression of chitinase-like protein-3 (YM1/Chi3l3) (also known as alternatively activated or M2 macrophages) and an anti-inflammatory state with expression of IL-10 (also known as M2-like macrophages)^8,15–17^.

Allergic asthma is characterized by the presence of high levels of IL-4 and IL-13 that can induce M2 polarisation and therefore, not surprisingly, we and others have found higher numbers of these macrophages in lungs of asthma patients and mice with allergic lung inflammation as compared to controls^7,8,11,14^. We also showed that the presence of higher numbers of these macrophages correlated with having worse symptoms^8^. In addition, transfer of IL-4/IL-13-stimulated macrophages to allergic mice aggravated the allergic inflammatory response in the lungs, suggesting that these macrophages contribute to the disease^7,9,10^. However, we have also shown that both human asthma as well as experimental asthma in mice is characterized by high numbers of IRF5+ M1 macrophages and low numbers of IL-10+ M2-like macrophages as compared to control^14^. In addition, having high numbers of IRF5+ M1 macrophages correlated with lower lung function, while having more IL-10+ M2-like macrophages correlated with better lung function^14^. Therefore a picture emerges of asthma being a disease in which homeostatic control of inflammation by IL-10+ M2-like macrophages is lost due to polarisation towards M1 and/or M2. This view is reinforced by studies showing less severe lung inflammation when adoptively transferring M2-like macrophages into the lungs of mice with allergic airway inflammation or studies steering M2 polarisation towards M2-like polarisation with the anti-inflammatory, innate immune plasma protein, serum amyloid P^9,13^.

Directing macrophage polarisation towards an anti-inflammatory M2-like behavior therefore appears to be a therapeutic option in asthma. A remaining question is whether inhibition of M2 polarisation per se will have a similar effect and will shift macrophage polarisation towards more anti-inflammatory behavior. To investigate this we inhibited M2 polarisation during and after development of HDM-induced lung inflammation by treating with cynaropicrin, a galectin-3 pathway inhibitor that potently inhibits M2 polarisation^18^.

## Results

### Treatment with cynaropicrin inhibits M2 macrophage polarisation

**Figure 1:**
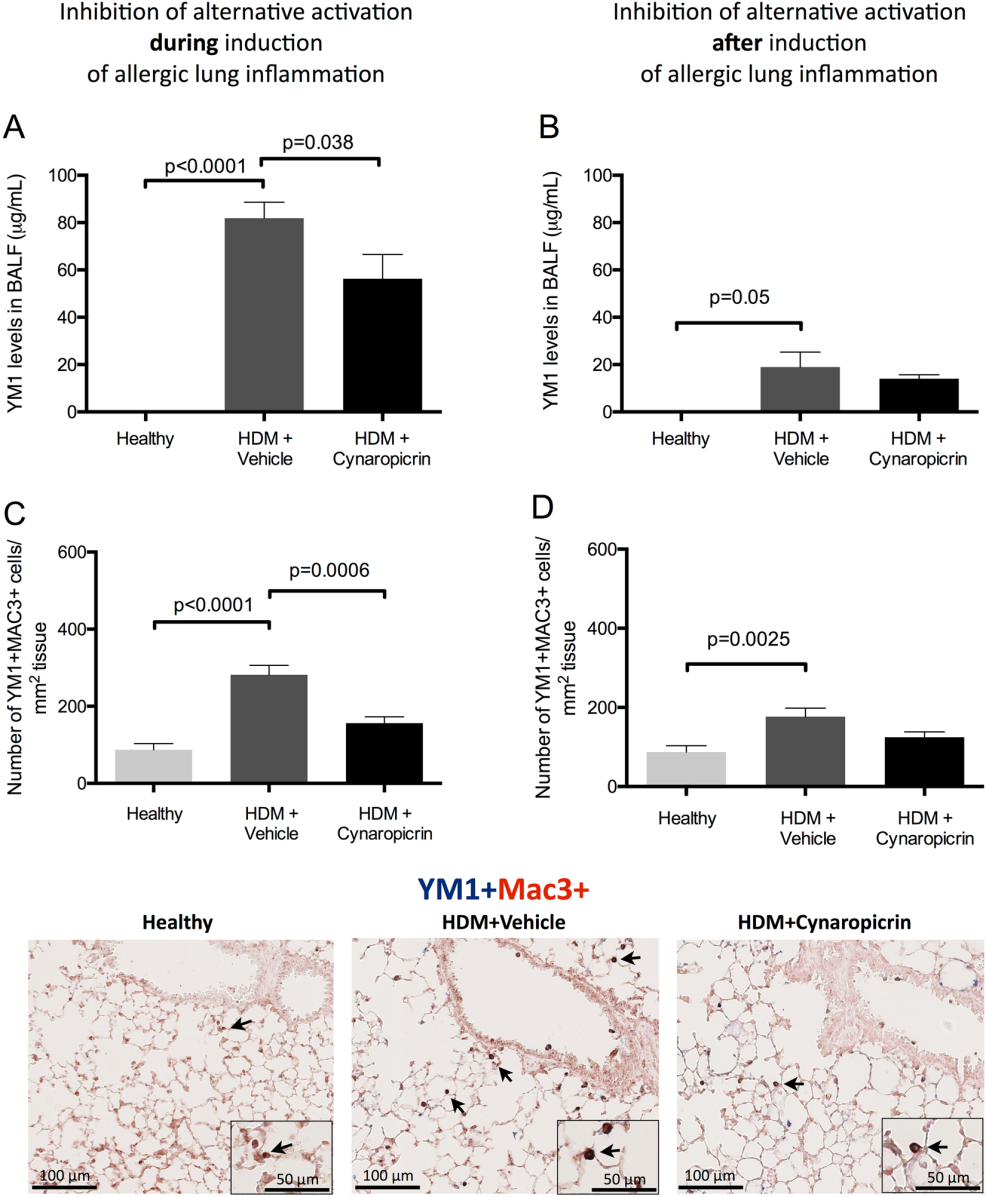
Treatment with cynaropicrin inhibits M2 macrophage polarisation. YM1 levels in BALF (**A** and **B**) of healthy mice (n = 8), vehicle-treated HDM-exposed mice (n = 16) and HDM-exposed mice treated with cynaropicrin (n = 8) during (left) and after (right) induction of allergic lung inflammation. The number of YM1 + Mac3 + macrophages (**C** and **D**) in lungs of healthy mice (n = 8), vehicle-treated HDM-exposed mice (n = 16) and HDM-exposed mice treated with cynaropicrin (n = 8) during (left) and after (right) induction of allergic lung inflammation. Representative pictures of the YM1 + Mac3 + double staining are shown in the bottom. Nuclear counter staining was not used to maximize visibility of double-positive cells (magnification 200x). Inserts are close up magnifications (magnification 400x) of double-positive cells. Significance was tested using a one-way ANOVA followed by Sidak’s multiple comparisons test comparing healthy vs. HDM + vehicle and HDM + vehicle vs. HDM + cynaropicrin.

To confirm previous findings, we tested the potential of cynaropicrin to inhibit polarisation into M2 macrophages. After two weeks or two weeks plus one day of HDM-exposures, the M2-related cytokine YM1 was found to be significantly higher in bronchoalveolar lavage fluid (BALF) as compared to PBS controls (Fig. 1A and B). Treatment of HDM-exposed mice with cynaropicrin resulted in lower YM1 levels in BALF compared to nontreated HDM-exposed mice, although this difference was only significant when treatment was started during induction of airway inflammation and not after induction.

To identify M2 macrophages in lung tissue, YM1 was stained in combination with a general macrophage marker (Mac3). More YM1+ macrophages were found in lungs of HDM-exposed mice than in lungs of healthy controls. Cynaropicrin-treatment resulted in significantly fewer YM1+ macrophages in lungs of mice treated during induction of allergic lung inflammation. Treatment of mice with established allergic lung inflammation showed a similar pattern in lowering YM1+ macrophage numbers, but the differences were not significant (Fig. 1C and D).

### Treatment with cynaropicrin dampens HDM-induced allergic lung inflammation

To assess the therapeutic potential of modulating YM1+ macrophages, we tested whether inhibition of M2 polarisation by cynaropicrin would affect induction of allergic lung inflammation and whether it would diminish an exacerbation in already established allergic lung inflammation. The presence of eosinophils, (MHCII-expressing) macrophages, T cell subsets and Th2-related cytokines was examined in lung tissue and BALF to assess allergic lung inflammation.

**Figure 2:**
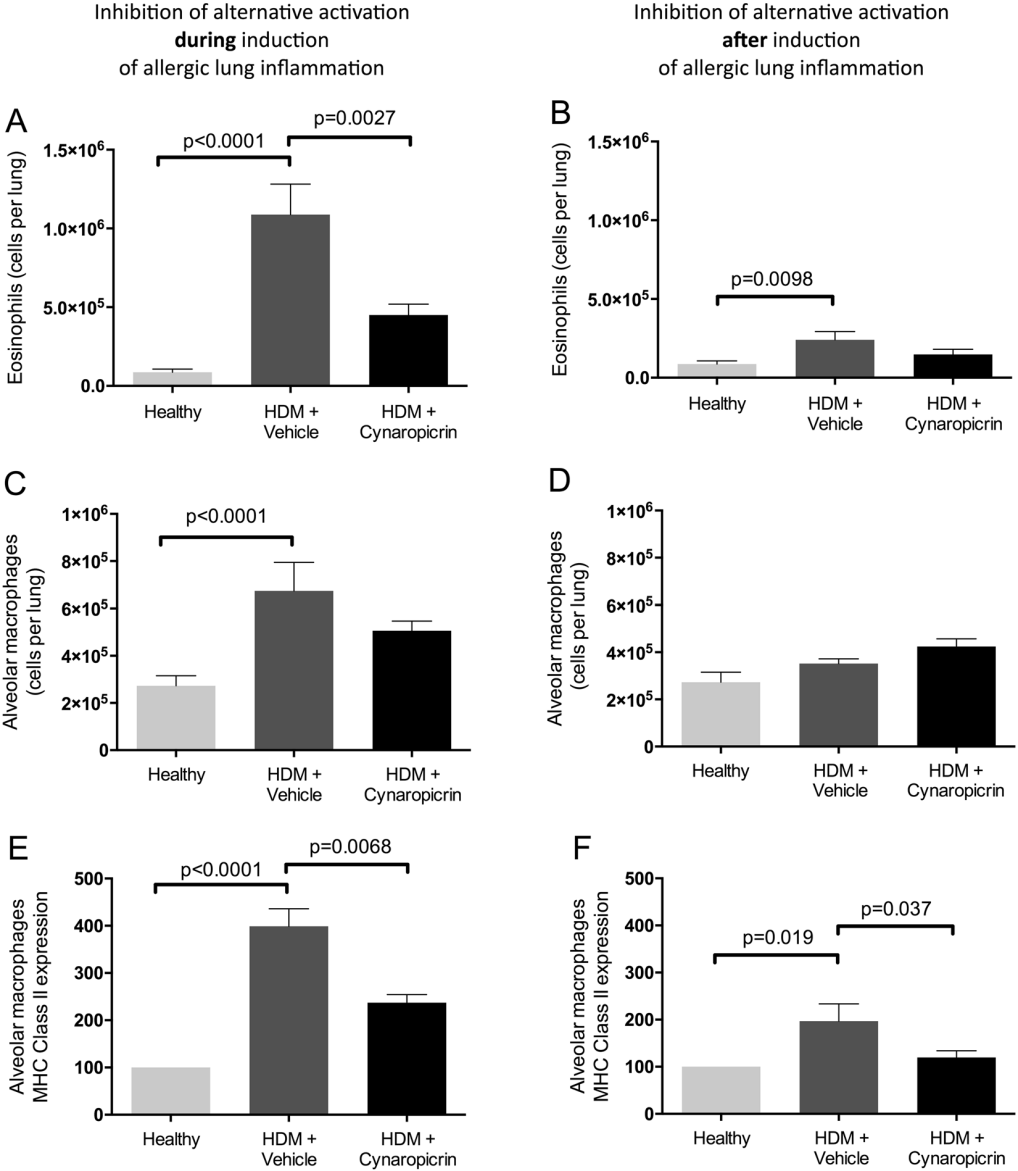
Cynaropicrin treatment dampens HDM-induced eosinophilic inflammation and macrophage activation. Number of eosinophils (**A** and **B**), numbers of alveolar macrophages (**C** and **D**) and expression of MHC class II on alveolar macrophages (**E** and **F**) in lungs of healthy mice (n = 8), vehicle-treated HDM-exposed mice (n = 16) and HDM-exposed mice treated with cynaropicrin (n = 8) during (left) and after (right) induction of allergic lung inflammation. Significance was tested using a one-way ANOVA followed by Sidak**’**s multiple comparisons test comparing healthy vs. HDM+ vehicle and HDM+ vehicle vs. HDM+ cynaropicrin.

HDM exposure induced higher numbers of eosinophils, total and MHCII-expressing macrophages (Fig. 2A,C and E), CD4+ T cells, effector T cells, regulatory T cells (Table 1) in lungs as compared to healthy controls. Inhibition of M2 polarisation during induction of allergic lung inflammation resulted in lower numbers of eosinophils and less expression of MHCII on macrophages. Total numbers of macrophages, CD4+ T cells, effector T cells and regulatory T cells were not affected by cynaropicrin treatment.

**Table 1:** T cells in lungs and BALF of healthy mice, vehicle-treated HDM-exposed mice and HDM-exposed mice treated with cynaropicrin during and after induction of allergic lung inflammation. Data are presented as mean ± standard error of the mean (SEM). Shown p values represent the comparison of healthy versus HDM+ vehicle-treated mice.

	Healthy	Inhibition of alternative activation during induction of allergic lung inflammation		Inhibition of alternative activation after induction of allergic lung inflammation	
		HDM+ vehicle	HDM+ Cynaropicrin	HDM+ vehicle	HDM+ Cynaropicrin
CD4+ T cells (10^5^ cells/lung)	2.57 ± 0.28	**8.89 ± 1.44 (p = 0.022)**	5.88 ± 0.51	**4.30 ± 0.35 (p = 0.0072)**	4.28 ± 0.36
Regulatory T cells (10^5^ cells/lung)	0.11 ± 0.02	**0.81 ± 0.16 (p = 0.022)**	0.47 ± 0.04	**0.43 ± 0.04 (p < 0.0001)**	0.45 ± 0.09
Effector T cells (10^5^ cells/lung)	0.38 ± 0.08	**1.47 ± 0.29 (p < 0.0001)**	1.06 ± 0.17	**0.93 ± 0.08 (p = 0.0001)**	0.96 ± 0.08

In mice with established allergic lung inflammation, that were treated with cynaropicrin afterwards, similarly low numbers of eosinophils were found in lungs as compared to vehicle-treated HDM-exposed mice (Fig. 2B). Total numbers of macrophages (Fig. 2D), CD4+ T cells, effector T cells and regulatory T cells (Table 1) were not affected by cynaropicrin treatment in established allergic lung inflammation, but macrophages were found to express less MHCII after treatment (Fig. 2F).

Higher levels of the M2 macrophage-associated cytokines IL-4 and IL-13 were found after two weeks of HDM-exposure as compared to healthy controls (Table 2

**Table 2:** Th2-related and anti-inflammatory cytokines in lungs of healthy mice, vehicle-treated HDM-exposed mice and HDM-exposed mice treated with cynaropicrin during and after induction of allergic lung inflammation. Data are presented as mean ± SEM. *Represents the comparison of healthy versus HDM+ vehicle-treated mice, while #represents the comparison of HDM+ vehicle versus HDM+ cynaropicrin-treated mice.

	Healthy	Inhibition of alternative activation during induction of allergic lung inflammation		Inhibition of alternative activation after induction of allergic lung inflammation	
		HDM+ vehicle	HDM+ Cynaropicrin	HDM+ vehicle	HDM+ Cynaropicrin
***Th2-related cytokines***					
IL-13 (pg/g lung tissue)	3.8 ± 2.2	7.3 ± 1.1 (*p = 0.044)	**5.0 ± 0.3 (#p = 0.044)**	2.9 ± 0.6	4.2 ± 0.7
IL-4 (pg/g lung tissue)	1.5 ± 0.6	3.3 ± 0.4 (*p = 0.0238)	2.8 ± 0.4	1.7 ± 0.3	2.3 ± 0.4
***Anti-inflammatory cytokines***					
IL-10 (pg/g lung tissue)	12.6 ± 1.4	9.1 ± 1.1 (*p = 0.09)	10.8 ± 1.7	9.5 ± 0.6 (*p = 0.07)	7.5 ± 0.8

). Cynaropicrin-treatment resulted in lower levels of IL-13 in lungs of mice that were treated during induction of allergic lung inflammation.

In mice with established allergic lung inflammation IL-4 and IL-13 levels were similar to those in lungs of healthy controls. Cynaropicrin treatment of mice with established allergic lung inflammation did not affect levels of IL-4 and IL-13 (Table 2).

### Treatment with cynaropicrin changes the balance in predominant macrophage phenotypes

Since cynaropicrin treatment inhibits M2 polarisation of macrophages, but total macrophage numbers were not affected, the presence of macrophage phenotypes was studied. To identify different macrophage phenotypes, lung tissue was dual stained for the Mac3 macrophage marker and either YM1, IRF5, or IL-10.

**Figure 3:**
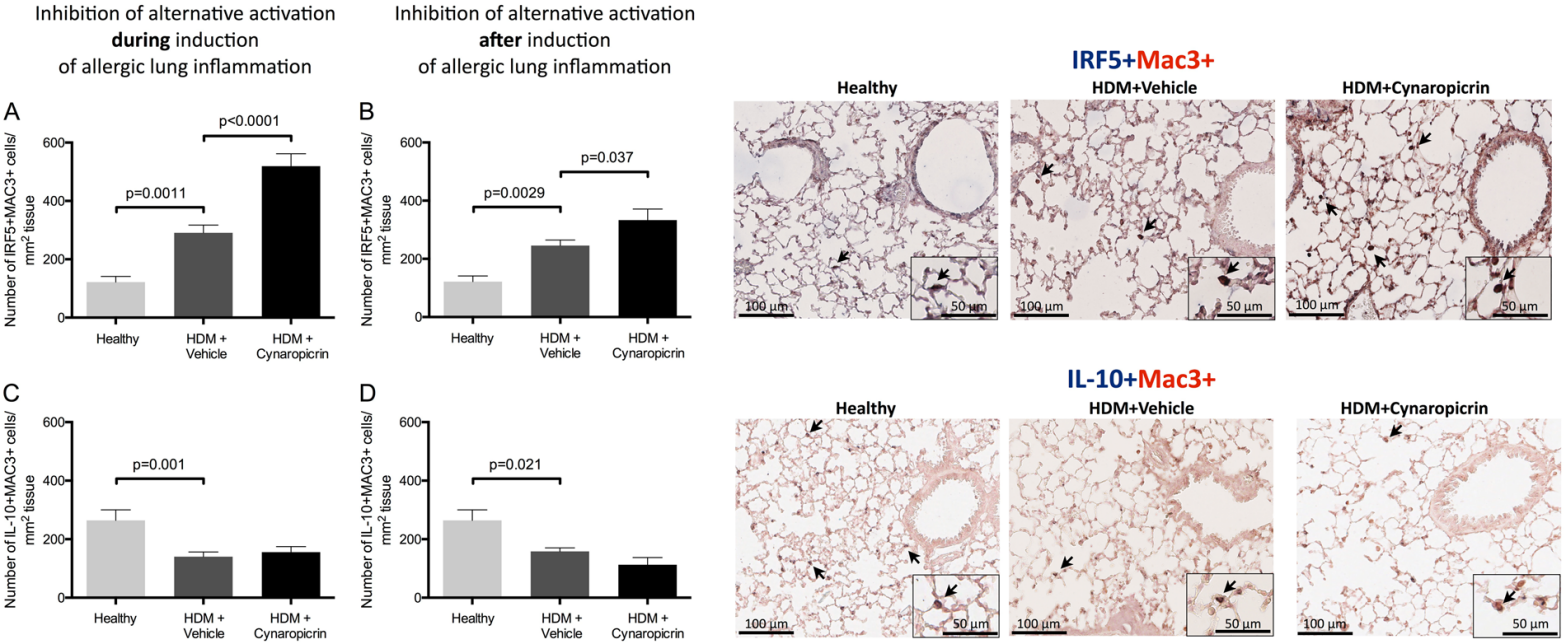
Cynaropicrin treatment changes the balance in macrophage phenotypes. The number of IRF5 + Mac3 + macrophages (**A** and **B**) and IL-10 + Mac3 + macrophages (**C** and **D**) in lungs of healthy mice (n = 8), vehicle-treated HDM-exposed mice (n = 16) and HDM-exposed mice treated with cynaropicrin (n = 8) during (left) and after (right) induction of allergic lung inflammation. Representative pictures of the IRF5 + Mac3 + and IL-10 + Mac3 + double-stainings are shown on the right. Nuclear counter staining was not used to maximize visibility of double-positive cells (magnification 200x). Inserts are close up magnifications (magnification 400x) of double-positive cells. Significance was tested using a one-way ANOVA followed by Sidak’s multiple comparisons test comparing healthy vs. HDM+ vehicle and HDM+ vehicle vs. HDM+ cynaropicrin.

As shown in Fig. 1, mice exposed to HDM had more YM1+ macrophages in lungs compared to healthy controls and cynaropicrin-treatment resulted in significantly lower numbers of YM1+ macrophages in lungs of mice treated during induction of allergic lung inflammation (Fig. 1C and D). Treatment of mice with established allergic lung inflammation showed a similar pattern in lowering YM1+ macrophage numbers, but the differences were not significant. Higher numbers of IRF5+ macrophages were observed after HDM-exposure as compared to healthy controls, but even more IRF5+ macrophages were found in mice treated with cynaropicrin during and after induction of allergic lung inflammation (Fig. 3A and B). Numbers of IL-10+ macrophages were lower in allergic lungs than in healthy lungs. Treatment of allergic mice with cynaropicrin during or after induction of inflammation did not affect the low numbers of IL-10+ macrophages (Fig. 3C and D). These results were supported by a trend towards lower levels of IL-10 that we found in lung tissue of HDM-treated mice in both models and the inability of cynaropicrin treatment to increase these levels again (Table 2).

### Treatment with cynaropicrin results in neutrophilic lung inflammation

**Figure 4:**
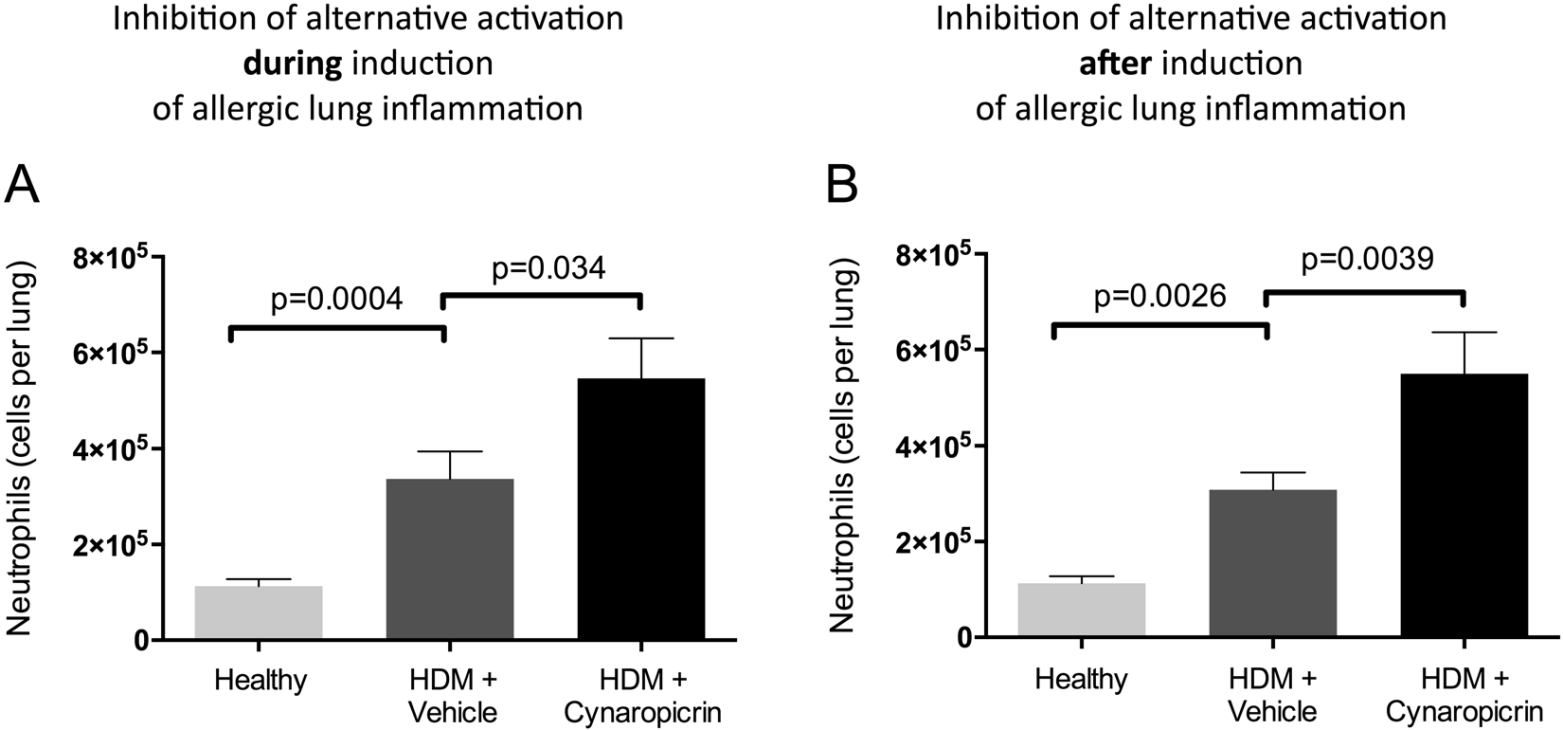
Cynaropicrin treatment results in neutrophilic inflammation. Number of neutrophils in lungs of healthy mice (n = 8), vehicle-treated HDM-exposed mice (n = 16**)** and HDM-exposed mice treated with cynaropicrin (n = 8) during (**A**) and after (**B**) induction of allergic lung inflammation. Significance was tested using a one-way ANOVA followed by Sidak’s multiple comparisons test comparing healthy vs. HDM+ vehicle and HDM+ vehicle vs. HDM+ cynaropicrin.

Cynaropicrin-treatment resulted in more IRF5+ macrophages, suggesting that a different type of inflammation is induced. We further studied the lung inflammation and found more neutrophils in lungs of allergic mice as compared to healthy controls and even more neutrophils were found in lungs of mice that were treated with cynaropicrin both during and after induction of allergic lung inflammation (Fig. 4A and B).

Furthermore, we observed higher levels of IRF5+ macrophage-related cytokines IL-12, IFNγ and TNFα in allergic lungs in comparison to healthy controls (Table 3

**Table 3:** Th1-and Th17-related cytokines in lungs of healthy mice, vehicle-treated HDM-exposed mice and HDM-exposed mice treated with cynaropicrin during and after induction of allergic lung inflammation. Data are presented as mean ± SEM. *Represents the comparison of healthy versus HDM+ vehicle-treated mice, while #represents the comparison of HDM+ vehicle versus HDM+ cynaropicrin-treated mice.

	Healthy	Inhibition of alternative activation during induction of allergic lung inflammation		Inhibition of alternative activation after induction of allergic lung inflammation	
		HDM+ vehicle	HDM+ Cynaropicrin	HDM+ vehicle	HDM+ Cynaropicrin
***Th1-related cytokines***					
IL-12 (pg/g lung tissue)	0.62 ± 0.07	1.11 ± 0.31 (*p = 0.0072)	**1.52 ± 0.19 (#p = 0.0393)**	0.77 ± 0.06	0.89 ± 0.10
IFNγ (pg/g lung tissue)	0.94 ± 0.15	1.60 ± 0.15 (*p = 0.044)	**2.52 ± 0.92 (#p = 0.044)**	1.38 ± 0.04 (*p = 0.06)	1.34 ± 0.09
TNFα (pg/g lung tissue)	2.1 ± 0.35	3.0 ± 0.25 (*p = 0.044)	**6.9 ± 2.8 (#p = 0.044)**	2.6 ± 0.27	2.8 ± 0.54
***Th17-related cytokines***					
IL-17 (pg/g lung tissue)	1.0 ± 0.38	2.3 ± 0.27 (*p = 0.0005)	1.78 ± 0.36	2.27 ± 0.67	3.4 ± 1.33

). Cynaropicrin-treatment during induction of allergic lung inflammation resulted in even higher levels of IL-12, IFNγ and TNFα as compared to HDM-exposed vehicle-treated mice. A similar pattern was seen when mice with established lung inflammation were treated with cynaropicrin, although these differences were not significant.

In view of the higher number of neutrophils found after cynaropicrin treatment, we also assessed levels of IL-17 in lung tissue (Table 3). Indeed, HDM-treatment resulted in higher levels of IL-17 as compared to healthy mice, but cynaropicrin treatment did not affect these levels any further. In addition, we did not see any correlations between IL-17 levels and neutrophil counts (data not shown).

### Treatment with cynaropicrin worsened airway hyperresponsiveness

Inhibition of M2 polarisation by cynaropicrin dampened eosinophilic lung inflammation, but induced neutrophilic lung inflammation. However, both eosinophilic and neutrophilic lung inflammation can be accompanied by airway hyperresponsiveness. Therefore, we tested whether airway hyperresponsiveness would be affected by cynaropicrin-treatment.

**Figure 5:**
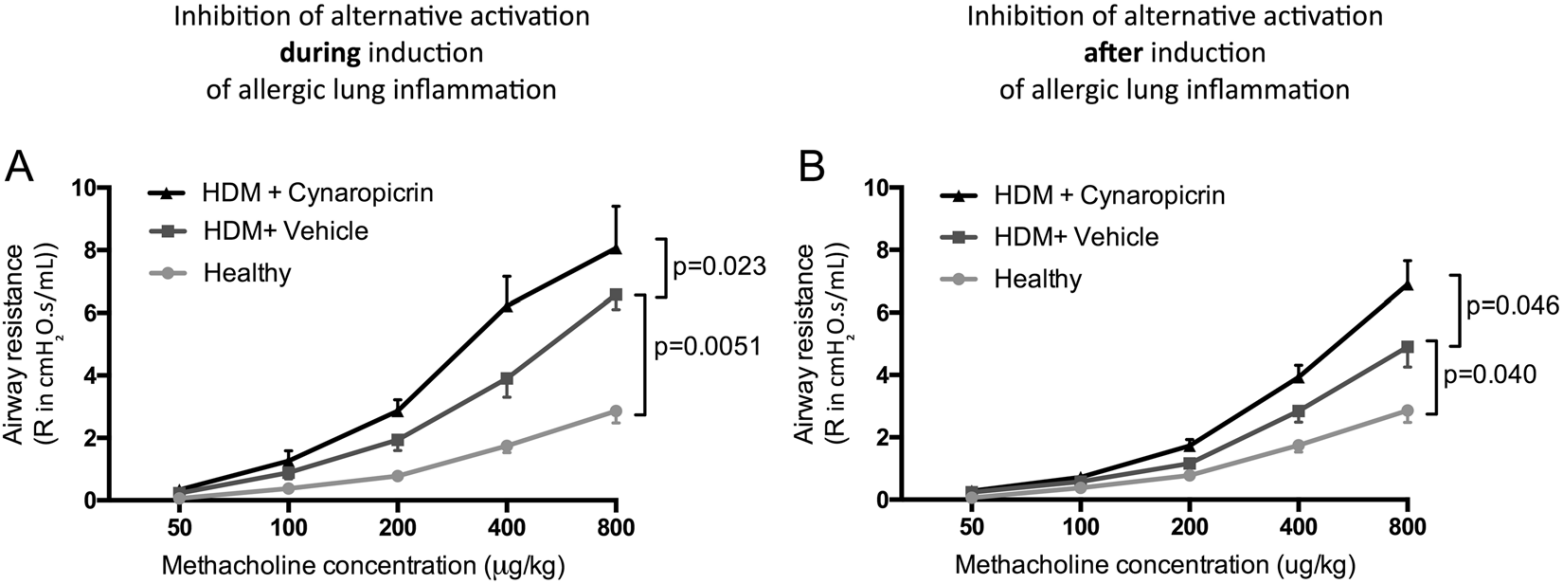
Cynaropicrin treatment worsened airway hyperresponsiveness. The dose-response resistance curve for methacholine of healthy mice (n = 8), vehicle-treated HDM-exposed mice (n = 16) and HDM-exposed mice treated with cynaropicrin (n = 8) during (**A**) and after induction (**B**) of allergic lung inflammation. Significance was tested using a one-way ANOVA followed by Sidak**’**s multiple comparisons test comparing the area under the curve of healthy vs. HDM+ vehicle and HDM+ vehicle vs. HDM+ cynaropicrin.

HDM exposure induced a dose-dependent increase in airway hyperresponsiveness to methacholine, which was significantly higher as compared to PBS-exposed controls (Fig. 5A and B). Cynaropicrin-treatment, both during and after induction of allergic lung inflammation, increased airway hyperresponsiveness to methacholine even further.

### Treatment with cynaropicrin results in less airway fibrosis

In order to shed some more light on the mechanism behind the worsening of airway hyperresponsiveness after inhibition of M2 polarisation, we studied the effect of cynaropicrin-treatment on airway remodeling and fibrosis.

**Figure 6:**
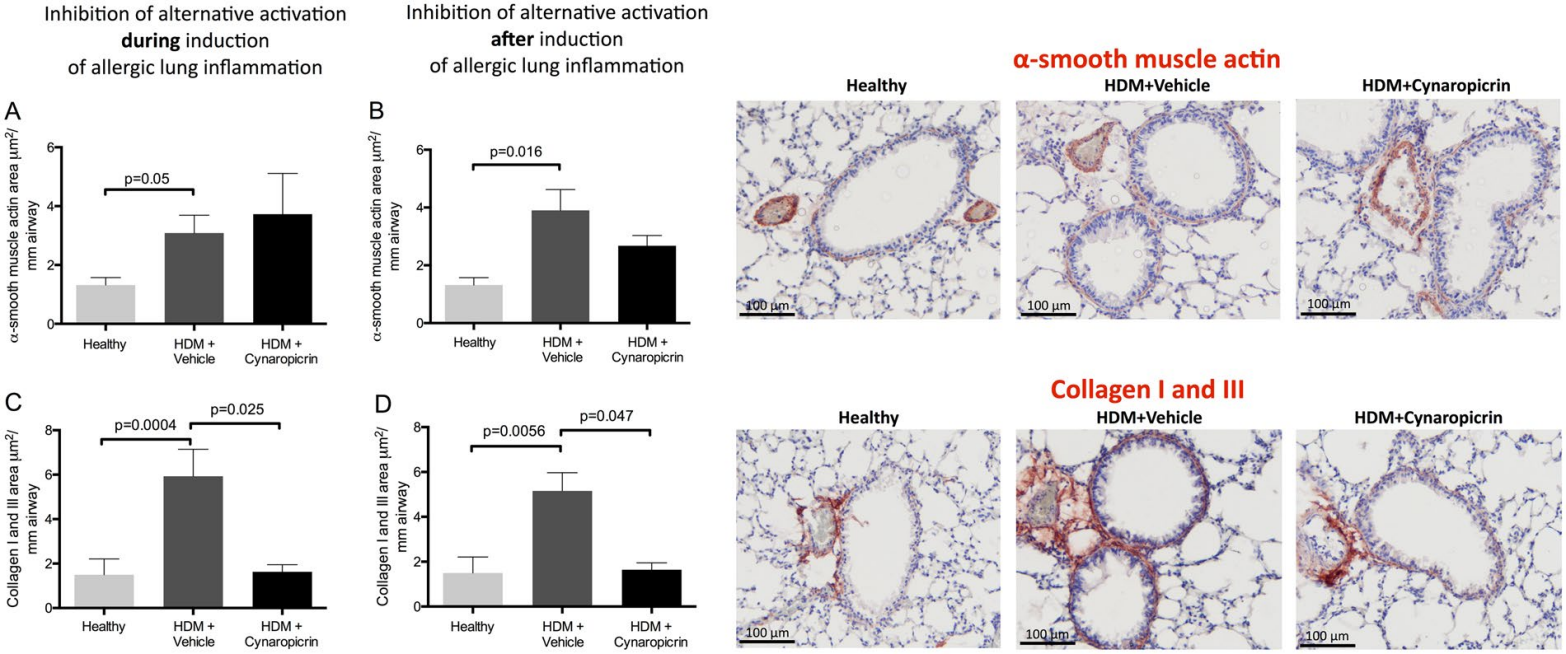
Cynaropicrin treatment results in less airway fibrosis. The area of α-smooth muscle actin (**A** and **B**) and collagen I and III (**C** and **D**) around the airways of healthy mice (n = 8), vehicle-treated HDM-exposed mice (n = 16) and HDM-exposed mice treated with cynaropicrin (n = 8) during (left) and after (middle) induction of allergic lung inflammation. Representative photos of α-smooth muscle actin and collagen I and III stainings with nuclear counter staining are shown (right panels, magnification 200x). Significance was tested using a one-way ANOVA followed by Sidak’s multiple comparisons test comparing healthy vs. HDM+ vehicle and HDM+ vehicle vs. HDM+ cynaropicrin.

Airways of HDM-exposed mice showed more remodeling than airways of control mice as reflected by a thicker layer of α-smooth muscle actin (α-SMA) and more collagen I and III deposition around the airways (Fig. 6A–D). Cynaropicrin-treatment both during and after induction of allergic lung inflammation did not affect the thickness of the α-SMA layer, but did result in significantly lower collagen deposition.

## Discussion

This study reveals an important dual role of YM1+ M2 macrophages in allergic lung inflammation. M2 macrophages are present in high numbers in lungs of humans and mice with allergic lung inflammation^7,8,11,12,19^, but their role in this disease remains controversial. By investigating the inhibition of M2 polarisation in mice during and after development of allergic lung inflammation, we found that YM1+ M2 macrophages are associated with both induction and progression of eosinophilic lung inflammation, but on the other hand prevent development of neutrophilic inflammation. In addition, YM1+ M2 macrophages contribute to airway remodeling, but thereby protect against the development of more severe airway hyperresponsiveness.

The treatment with cynaropicrin, an inhibitor of M2 polarisation, during and after induction of allergic lung inflammation dampened eosinophilic lung inflammation. These findings further substantiate previous findings that showed participation of M2 macrophages in T helper 2-mediated inflammatory diseases. We showed that the transfer of *in vitro* differentiated M2 macrophages into the airways of male asthmatic mice aggravated lung inflammation^7^. A study by Ford *et al*. showed that intraperitoneal injection of IL-4Rα-positive macrophages was sufficient to increase the allergic inflammatory response in the lung^10^. In a model of fungus-induced allergic airway disease, Moreira *et al*. showed that transfer of IL-4/IL-13-stimulated macrophages into the lungs of mice enhanced both inflammation and collagen deposition as compared to asthmatic mice not treated with these macrophages^9^. In contrast, Nieuwenhuizen *et al*. demonstrated that M2 macrophages are not necessary for allergic airway disease and may only be an association as a result of the elevated Th2 response^20^. They showed that airway hyperresponsiveness, remodeling and eosinophilic inflammation were not affected by decreased development of M2 macrophages by using LysM^cre^ mice with macrophage-restricted IL-4 receptor-α (IL-4Rα)-deficiency. Interestingly, recent data showed that these LysM^cre^ mice successfully abrogate IL-4Rα signaling on mature tissue resident macrophages, but fail to delete this on more immature macrophages arising from proliferation or from recruited monocyte precursors^21^. In addition, it was found that M2 macrophages derived from monocytes or from tissue macrophages are phenotypically and functionally distinct^22^. Combining these findings, it seems likely that in particular M2 macrophages that are newly derived through either proliferation or from recruited monocytes, are the active contributors to the disease rather than the mature resident M2 macrophages. The use of the pharmacological agent cynaropicrin is an advantage in this study as this can inhibit M2 polarisation of both newly derived and resident macrophages, as opposed to the use of knockout or overexpression models, which target one macrophage population only.

Our method of inhibiting alternative activation involved intranasal administration of cynaropicrin, a galectin-3 pathway inhibitor. Activation of the galectin-3/CD98/PI3K-pathway has previously been shown to be required for polarisation into YM1+ M2 macrophages. Cynaropicrin specifically inhibits the development of M2 macrophages and does not affect other macrophage phenotypes as shown by Mackinnon *et al*.^18^. We confirmed their findings with preliminary *in vitro* and *in vivo* studies and in addition ruled out possible effects of toxicity (data not shown). It is, however, unknown how *in vivo* administration of cynaropicrin influences other cells in the lung that express galectin-3. In basal conditions a variety of cells express galectin-3, including macrophages, eosinophils, dendritic cells (DCs), and epithelial cells^23,24^. The current study did not investigate the direct impact of cynaropicrin on eosinophils but it cannot be ruled out that the lower numbers we found upon cynaropicrin treatment may have been caused by inhibition of rolling, adhesion and migration of eosinophils as was shown in a galectin-3 knockout model^25^. Neutrophils do not express galectin-3 and therefore we do not expect that the higher neutrophil numbers can be explained by a direct effect of cynaropicrin on neutrophils^26^. Galectin-3 in epithelial cells enhances proliferation and therefore intranasally administered cynaropicrin could affect proliferation of airway epithelium^27^. We did not investigate whether proliferation of epithelial cells was affected by cynaropicrin treatment, but we did study whether IL-33 production (important for the induction of Th2 responses) by type II alveolar epithelial cells was affected by cynaropicrin treatment and we found no effects of cynaropicrin (data not shown). In addition, we did not observe any obvious epithelial changes in the histological sections of lung tissue. Furthermore, the expression pattern of galectin-3 is different in an inflammatory environment such as lungs of allergic mice^28^. In lung tissue of these mice it was shown that macrophages are the major cell type expressing galectin-3. This may suggest that intranasal cynaropicrin treatment will mostly affect macrophages in mice with HDM-induced inflammation.

Even though cynaropicrin does not directly induce polarisation into other macrophage phenotypes, we found higher numbers of IRF5+ M1 macrophages, more neutrophils and higher levels of IL-12 in allergic mice that were treated with cynaropicrin as compared to untreated allergic mice. Other studies have found similar changes. Nieuwenhuizen *et al*. showed this shift towards the induction of IRF5+ M1 macrophages after blocking signaling for M2 polarisation^20^. Similarly, Hong *et al*. showed that ovalbumin sensitization and challenge of IL-4 receptor knockout mice increased the number of IFNγ- and TNFα- producing macrophages and neutrophilic inflammation^29^. Thus, our data and those of others suggest that YM1+ M2 macrophages contribute to eosinopilic lung inflammation and that inhibition of M2 polarisation during HDM exposures results in a skewing towards M1 polarisation of macrophages accompanied by neutrophilic inflammation. Therefore, inhibiting M2 polarisation as a therapeutic strategy for asthma does not appear to be a beneficial approach as neutrophilic asthma is generally more difficult to treat in the clinic. Interestingly, in IRF5−/− mice an opposite effect was demonstrated by Oriss *et al*. using a mouse model of severe asthma^30^. Although IRF5−/− mice subjected to the severe asthma model displayed lower IFNγ and IL-17 responses and lower neutrophil numbers, they had higher Th2 responses with higher eosinophil numbers. Taken together, these data suggest that the type of macrophages present in lung tissue may influence the direction of inflammation in asthma. We did not specifically examine the cytokine/chemokine expression by macrophages in this study, but the data suggest that macrophages actively contributed to the recruitment of either eosinophils or neutrophils to the site of inflammation. This is reinforced by the fact that YM1+ M2 macrophages secrete copious amounts of YM1 that has been shown to be a chemotactic factor for eosinophils and IRF5 expression has been shown to control neutrophil chemokine expression^31,32^. Consequently, the clinical implications of these combined results are that inhibition of either neutrophil-or eosinophil-associated macrophage phenotypes is not a valid therapeutic option as this may result in domination of the other. The only promising therapeutic option that may be investigated for clinical applications therefore appears to be increasing the number of IL-10+ M2-like macrophages as we and Moreira *et al*. have shown before^9,13^.

An interesting observation in our study is the apparent disconnect between airway hyperresponsiveness and the amount of collagen remodeling around the airways. Airway hyperresponsiveness is associated with airway smooth muscle hyperplasia, as demonstrated in this study and by others, by a thicker α-SMA layer and more collagen deposition around the airways^33–35^. Our data now demonstrate that this collagen deposition appears to protect the airways from even worse contractility. This notion has been coined before and it was considered that airway remodeling might actually protect against airway hyperresponsiveness^36,37^. Although many studies show a correlation between airway remodeling and asthma severity, on basis of mathematical modeling assessing the effects of airway wall thickening on airway resistance it is unlikely that airway wall thickening can increase airway resistance to the extent observed in asthma patients^38^. In fact, some studies also found that airway reactivity was inversely correlated with airway wall thickness^36,39^. A study that compared males and females with allergic airway inflammation had similar findings as we had. The males had lower collagen content in the lungs than females, but they found that airway hyperresponsiveness to methacholine was higher in males as compared to females^40^. The authors concluded that the airway hyperresponsiveness is influenced by additional mechanisms, which are not necessarily related to remodeling, such as vagal reflex pathways. Our data, however, suggest that collagen deposition around the airways, which may contribute to stiffening, can protect against worsening of airway hyperresponsiveness.

As YM1+ M2 macrophages are associated with fibrosis, the decrease in collagen deposition may be directly linked to the inhibition of M2 polarisation^41,42^. Therefore, we measured TGF-β levels, as an important mediator produced by profibrotic macrophages and a major stimulator of collagen production in the lung tissue, but there were no differences between the untreated and treated HDM-induced allergic mice (data not shown)^43^. Another explanation for less collagen in our model could be the high presence of IRF5+ macrophages and its association with resolution of fibrosis through production of matrix metalloproteinases that can degrade collagens^44^.

In conclusion, this study demonstrates an important dual role for YM1+ M2 macrophages in the induction and progression of allergic lung inflammation. YM1+ M2 macrophages are associated with both induction and progression of eosinophilic lung inflammation and airway remodeling. However, inhibition of M2 polarisation as a therapeutic intervention for asthma has unfortunate consequences as this shifted the balance towards more IRF5+ M1 macrophages and neutrophilic lung inflammation and the development of more severe airway hyperresponsiveness.

## Materials and Methods

### Animals

Specific pathogen free female BALB/c mice (6–8 weeks old) were purchased from Harlan (Horst, The Netherlands). The mice were kept in a temperature and light-controlled room and were housed in groups of 4. There was *ad libitum* access to water and food. The University of Groningen’s institutional Animal Care and Use Committee approved our animal protocol (application number 6272C). All methods were carried out in accordance with relevant national and local guidelines and regulations regarding the use of experimental animals and proper research conduct.

### HDM model and inhibition of alternative activation of macrophages

**Figure 7:**
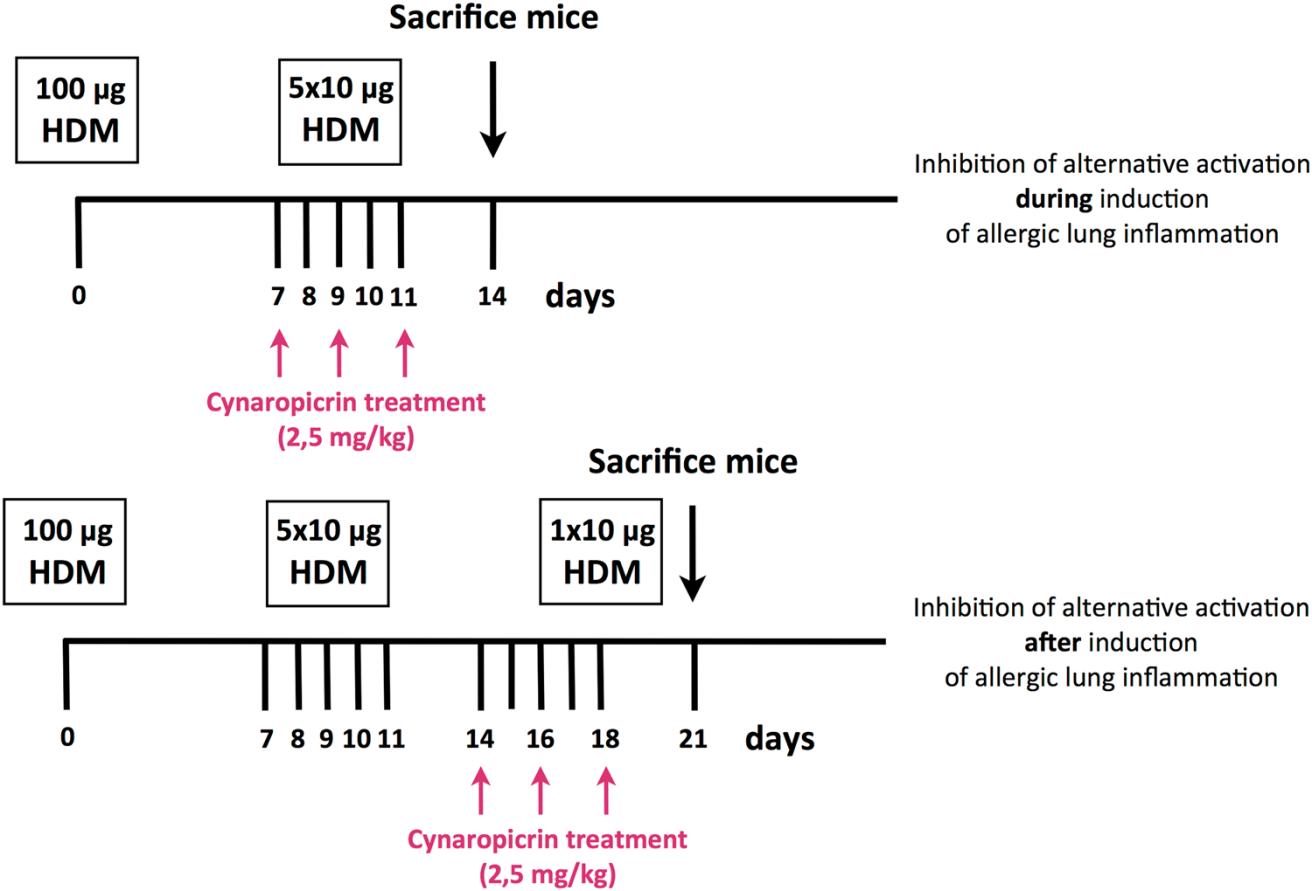
Experimental design of the study.

Female BALB/c mice (n = 16 per group) were exposed intransally to whole body HDM extract *Dermatophagoides Pteronyssinus* (Greer laboratories, Lenoir, USA) according to our established 14-day model^11^. Mice received 100 μg HDM in 40 μl PBS on day 0 and 10 μg HDM in 40 μl PBS on day 7–11 intranasally under isoflurane anesthesia to induce allergic lung inflammation. Mice exposed to 40 μl PBS according to the HDM protocol served as healthy controls. To inhibit M2 polarisation of macrophages during induction of lung inflammation mice were exposed intranasally to cynaropicrin (2.5 mg/kg in 35 μl 4% DMSO/PBS, BioNaturis, Jerez de la Frontera, Spain). In a preliminary dose-finding study using 10, 5 and 2.5 mg/kg cynaropicrin, we found that 2.5 mg/kg inhibited M2 polarisation without signs of toxicity. Cynaropicrin was administered on day 7, 9 and 11 right before HDM administration. To study inhibition of M2 polarisation of macrophages after induction of lung inflammation mice received cynaropicrin on days 14, 16 and 18. On day 18, these mice received an exacerbation dose of 10 μg HDM in 40 μl PBS. Control mice received treatment with either 35 μl 4% DMSO/PBS or 35 μl PBS but since no significant effect of 4% DMSO was observed these control groups were combined. Thus *per readout*, three experimental groups were studied per model (14 days and 21 days): Healthy/PBS-exposed (n = 8), HDM+ vehicle-exposed (n = 16) and HDM+ cynaropcrin-exposed (n = 8). Three days after the last exposure the mice were sacrificed. The experimental study design is shown in Fig. 7.

On the day of sacrifice, the lungs of 8 out of 16 mice were lavaged to collect bronchoalveolar lavage fluid (BALF). Their left lung lobe was digested for flow cytometry purposes and their right lung lobe was used for cytokine analyses. The other 8 mice were used to determine airway hyperresponsiveness and these mice were then sacrificed for histological analyses of lung tissue.

### Bronchoalveolar lavage fluid (BALF)

BALF was collected by gently flushing the lungs with 1 ml of PBS three times and BALF supernatants were used to determine the levels of YM1 by ELISA kit (R&D Systems, Oxon, UK) strictly following the manufacturer’s instructions.

### Lung digestion

The left lung lobe was cut into small pieces and incubated in digestion buffer for 45 min at 37 °C in a shaking water bath. The digestion buffer was RPMI 1640 medium (Lonza, Verviers, Belgium) containing 10% fetal calf serum (Lonza), 0.7 mg/ml collagenase A (Sigma-Aldrich, Zwijndrecht, The Netherlands) and 10 μg/ml DNAse I (grade II from bovine pancreas, Roche Applied Science, Almere, Netherlands). After digestion, the lung tissue was forced through a 70 μm nylon strainer (BD Biosciences, Breda, Netherlands) to obtain single lung cell suspensions. A 2-minute incubation with 10 times diluted Red Blood Cell lysis buffer (Biolegend, Fell, Germany) was done to lyze erythrocytes followed by centrifugation through 70 μm strainer caps. Cells were counted using a Casy cell counter (Roche Innovatis AG) and were ready for flow cytometric stainings.

### Flow cytometric analysis

T-cell subsets, macrophages, eosinophils, and neutrophils were examined by flow cytometry in single cell suspensions using two different cocktails of antibodies.

Frequencies of effector T cells (CD3^+^CD4^+^CD25^+^Foxp3^**−**^) and regulatory T cells (CD3^+^CD4^+^CD25^+^Foxp3^+^) were identified using anti-CD3-APC/Cy7 diluted 1:100 (Biolegend, Fell, Germany, catalog number (cat#) 100221), anti-CD4-PE/Cy7 diluted 1:100 (Biolegend, cat# 100421), anti-CD25-PE diluted 1:100 (Biolegend, cat# 102008), and anti-Foxp3-FITC diluted 1:100 (eBioscience, Vienna, Austria, cat# 11-5773-82). An isotype control was used for the Foxp3 staining diluted 1:100 (rat IgG2ak-FITC, eBioscience, cat# 11-4321-42).

Frequencies of alveolar macrophages (autofluorescence^+^F4/80^+^CD11c^+^), activated alveolar macrophages (autofluorescence^+^F4/80^+^CD11c^+^MHCII^+^), eosinophils (autofluorescence^−^F4/80^−^GR1^−^MHCII^−^) and neutrophils (autofluorescence^−^F4/80^−^GR1^+^) were identified by using autofluorescence in the FITC channel, anti-CD11c-APC/Cy7 diluted 1:50 (Biolegend, cat# 117324), anti-CD11b-PerCP/Cy5.5 diluted 1:200 (Biolegend, cat# 101227), anti-MHC class II-Alexa Fluor 700 diluted 1:250 (Biolegend, cat# 107622), anti-F4/80-Pacific Blue diluted 1:25 (Biolegend, cat# 123124), and anti-GR1-PE/Cy7 diluted 1:200 (Biolegend, cat# 108415).

Approximately 1 × 10^6^ lung cells were incubated for 30 minutes in the dark on ice with the appropriate antibody mix including 1% normal mouse serum. After subsequent washing of the cells with PBS containing 2% FCS and 5 mM EDTA (PFE), cells were incubated with FACS lysing solution (BD Biosciences) on ice for 30 minutes. Cells were then washed twice with PFE, and resuspended in PFE and kept in the dark on ice until flow cytometric analysis. The cells stained for T-cell subsets were (instead of incubation with FACS lysing solution) incubated for 30 minutes with fixation and permeabilization buffer (eBioscience). After that, cells were washed with permeabilization buffer. Subsequently, the cells were incubated for 30 minutes with anti-Foxp3 and afterwards washed twice with permeabilization buffer. Cells were resuspended in PFE and kept in the dark at 4 degrees Celsius until flow cytometric analysis. Scatter and fluorescence were measured on a LSR-II flow cytometer (BD Biosciences) and data were analysed using FlowJo Software (Tree Star, Ashland, USA). Examples or our gating strategies can be found in supplemental Figures S1 and S2.

### Lung homogenates and cytokines analysis

A Mini-Beadbeater (Biospec, Bartlesville, OK, USA) was used to homogenise snap frozen lung tissue for 45 seconds in 50 mM Tris-HCl buffer, supplemented with 150 mM NaCl, 0.002% Tween-20 (pH 7.5) and a Complete Mini Protease Inhibitor Cocktail tablet (1 tablet/10 mL, Sigma Aldrich). To remove insoluble material from the homogenates, a 30-minute spin at 12000 × g for 30 minutes was performed. Supernatants were stored at −80 °C until further analysis. Levels of IL-4, IL-10, IL-12, IL-13, IL-17 and TNFα and IFNγ were measured in lung supernatants by multiplex ELISA (eBioscience).

### Airway hyperresponsiveness

Airway hyperresponsiveness was assessed as described previously^45^. Briefly, anesthesized (ketamine/domitor) mice were cannulated tracheally and intravenously via the jugular vein. Mice were attached to a computer-controlled small-animal ventilator (Flexivent; SCIREQ, Montreal, Quebec, Canada) and ventilated at a breathing frequency of 280 breaths/min and a tidal volume of 10 ml/kg, which was pressure-limited at 300 mm H_2_O. Airway resistance (R in cmH_2_O.s/mL) in response to increasing doses of intravenously administered methacholine (acetyl-b-methylcholine chloride, Sigma-Aldrich) was calculated from the pressure response to a 2-s pseudorandom pressure wave.

### Histology

Both lungs were carefully filled with 50% Tissue-Tek® O.C.T.™ compound in PBS (Sakura, Finetek Europe B.V., Zoeterwoude, The Netherlands) through a cannula inserted in the trachea. The right lung was fixed in zinc (JB fixative^46^) and the left lung was fixed in formalin and both were thereafter embedded in paraffin.

The different macrophage subsets were determined in 3 µm formalin-paraffin sections using standard immunohistochemical procedures. Mac3 (anti-Mac3 diluted 1:50, BD Biosciences, cat# 550292), a general macrophage marker, was used in combination specific markers for different phenotypes. Numbers of IRF5+ macrophages were determined by a dual stain using Mac3 and IRF5 (anti-IRF5 diluted 1:75, ProteinTech Europe, Manchester, UK, cat# 10547-1-AP). YM1+ macrophages were identified by a dual stain using Mac3 and YM1 (anti-mECF-L diluted 1:50, R&D Systems, Oxon, UK, cat# AF2446), and IL-10+ macrophages were identified by a dual stain using Mac3 and IL-10 (anti-IL-10 diluted 1:25, Hycult, Uden, The Netherlands, cat# HP9016). Only double-positive cells in parenchymal lung tissue were counted. Numbers were corrected for the total surface area of lung tissue as measured by Aperio ImageScope viewing software 11.2.0.780 (Aperio, Vista, USA).

Airway fibrosis and remodeling was assessed by presence of collagen I/III or α-smooth muscle actin. Zinc-fixed paraffin sections were stained with anti-type I collagen antibody diluted 1:75 in combination with anti-type-III collagen antibody diluted 1:500 (both SouthernBiotec, Birmingham, AL, USA, cat# 1310-01 and 1330-01 respectively) or anti-α-smooth muscle actin antibody (α-SMA diluted 1:500, Sigma-Aldrich, cat# A2547) respectively. Collagen I and III around the airways and the α-SMA-positive layer directly adjacent to the airway epithelium were quantified in a total lung section by morphometric analysis. The surface of the positively stained area was expressed as μm^2^ per mm airway in the total lung section. Staining in the parenchyma and directly adjacent to blood vessels was excluded.

### Statistical Analysis

Results are presented as mean ± standard error of the mean. To verify whether data were normally distributed a D’Agostino & Pearson omnibus normality test was used. When data were not normally distributed they were log transformed to obtain a normal distribution. A one-way ANOVA followed by Sidak’s multiple comparisons test was used to compare healthy vs. HDM+ vehicle and HDM+ vehicle vs. HDM+ cynaropicrin. Differences in airway resistance were tested by comparing the area under the curve of healthy vs. HDM+ vehicle and HDM+ vehicle vs. HDM+ cynaropicrin. Differences were considered statistically significant when the P-value was lower than 0.05.

### Data availability statement

The datasets generated during and/or analysed during the current study are available from the corresponding author upon reasonable request.

## Electronic supplementary material

Supplementary file
