# Supplementary material for: Dual role of YM1+ M2 macrophages in allergic lung inflammation
Source: Sci Rep. 2018 Mar 23;8:5105. doi: 10.1038/s41598-018-23269-7 (PMC5865212; doi:10.1038/s41598-018-23269-7)
Supplement: Supplementary file 1 — Supplementary file [file 41598_2018_23269_MOESM1_ESM.pdf]

Online supplement to:

Dual role of YM1+ M2 macrophages in allergic lung inflammation

Christina Draijer[1, 2], Patricia Robbe [2,3], Carian E. Boorsma[1,2], Machteld N. Hylkema[2,3], Barbro N. Melgert[1,2]

[1]*University of Groningen, Department of Pharmacokinetics, Toxicology and Targeting, Groningen, The Netherlands*

[2]*University of Groningen, University Medical Center Groningen, GRIAC- Groningen Research Institute for Asthma and COPD, Groningen, The Netherlands*

[3]*University of Groningen, University Medical Center Groningen, Department of Pathology, Groningen, The Netherlands*

Corresponding author:

Prof. Dr. B.N. Melgert

Department of Pharmacokinetics, Toxicology and Targeting

University of Groningen

Antonius Deusinglaan 1

9713 AV Groningen

The Netherlands

Tel: +31-50-3632947

Fax: +31-50-3633247

Email: b.n.melgert@rug.nl

Supplemental figures

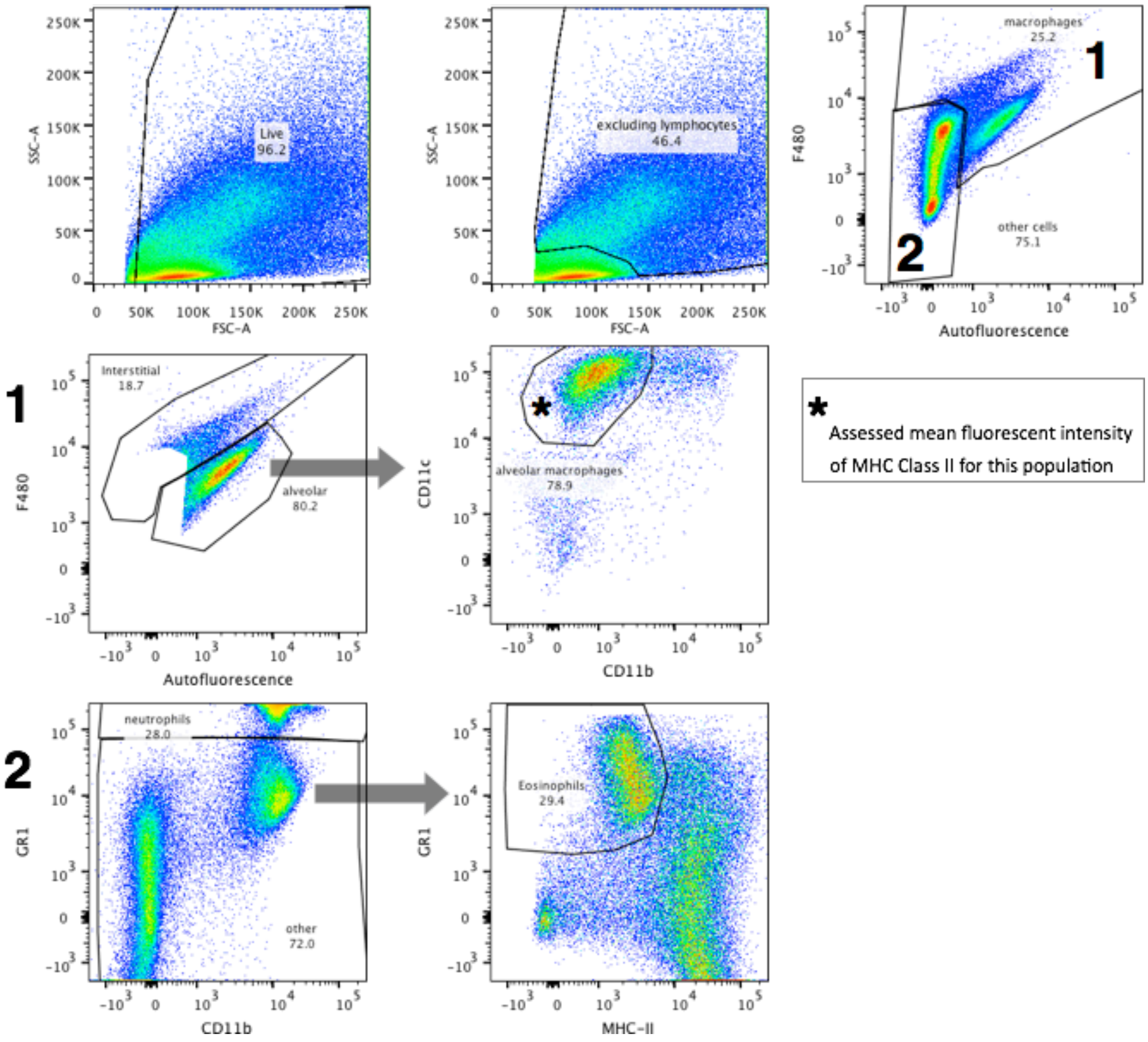

Figure S1. Gating strategy of macrophages, neutrophils, and eosinophils.

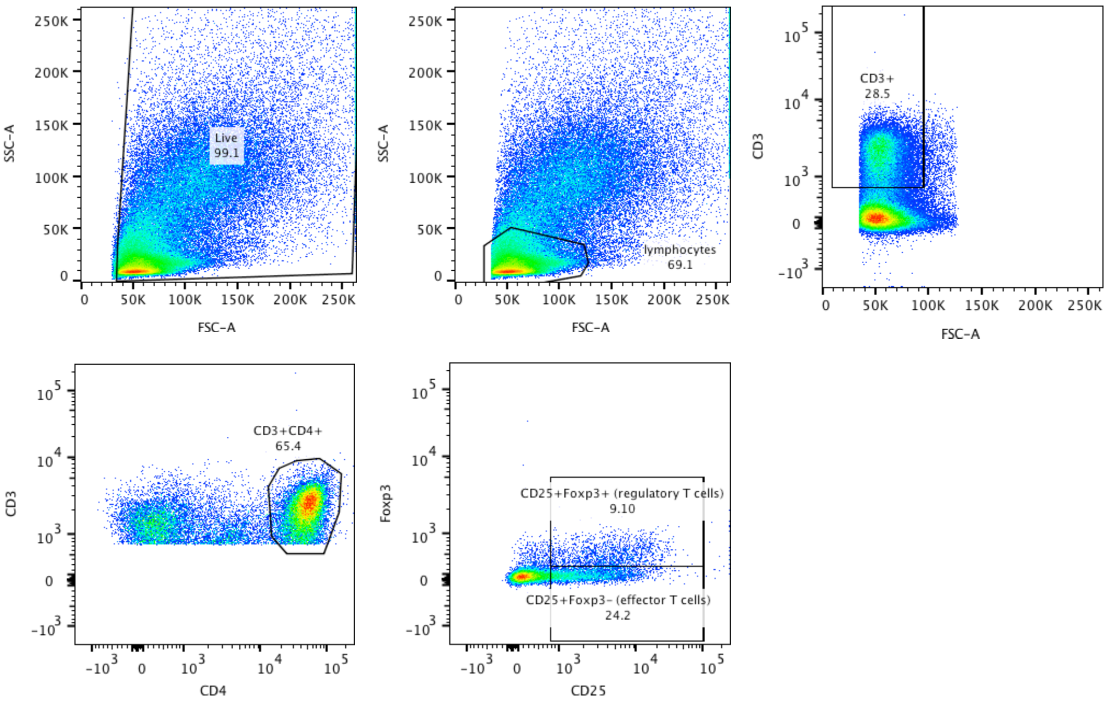

Figure S2. Gating strategy of CD4+ T cells, effector T cells and regulatory T cells.
